# The Role of Compulsivity in Body-Focused Repetitive Behaviors

**DOI:** 10.1016/j.jpsychires.2022.05.001

**Published:** 2022-05-05

**Authors:** Jon E. Grant, Samuel R. Chamberlain

**Affiliations:** a Department of Psychiatry & Behavioral Neuroscience, University of Chicago; b Department of Psychiatry, Faculty of Medicine, University of Southampton, UK; and Southern Health NHS Foundation Trust, Southampton, UK

**Keywords:** compulsivity, trichotillomania, skin picking, psychosocial functioning

## Abstract

Trichotillomania (TTM) and skin picking disorder (SPD) have been characterized as body-focused repetitive behaviors (BFRBs) and have been grouped in the DSM-5 as part of the obsessive-compulsive and related disorders. Despite the clinical similarities between BFRBs and the compulsions of OCD, there has been little research regarding the degree to which compulsivity underpins these behaviors. 91 adults (69 female; mean age 30.9 years) with DSM-5 TTM or SPD completed standard diagnostic interviews, basic demographic information, symptom inventories about TTM and SPD, and measures of disability/functioning. Compulsivity was measured using the 15-item Cambridge-Chicago Compulsivity Trait Scale (CHI-T). Relationships between CHI-T scores and the other measures of interest were characterized using Pearson’s correlations. Of the 91 participants, 48 (54.6%) had TTM, 37 (42.0%) had SPD and 2 (3.4%) had both. CHI-T total scores correlated with worse disability and quality of life but not with BFRB symptom severity. This study demonstrates that compulsivity is a marked contributor to worse disability and quality of life in people with BFRBs, more so than conventional measures of BFRB symptom severity (which did not correlate with disability/quality of life). Future work should include compulsivity measures in BFRB studies (including treatment trials) and explore its role in other related disorders.

## Introduction

Trichotillomania (TTM) and skin picking disorder (SPD) have been grouped together as body-focused repetitive behaviors (BFRBs) and included in the Diagnostic and Statistical Manual Version 5 (DSM-5) as part of the obsessive-compulsive and related disorders (American Psychiatric Association, 2013). Usually lacking an obsessional component (Christenson et al., 1991; Swedo and Leonard, 1992), BFRBs are characterized by repetitive behaviors, that are oftentimes preceded by negative internal states, impaired response inhibition (Chamberlain et al., 2006), and negative consequences such as alopecia or excoriations (Stanley et al., 1995; Grant et al., 2007). Despite the clinical similarities between BFRBs and the compulsions of OCD, and the symptoms of BFRBs being suggestive of compulsivity, there has been little research regarding the degree to which compulsivity underpins these behaviors (Figee et al., 2016). The concept of compulsivity is highly relevant to understanding BFRBs, including from clinical and biological perspectives. Compulsivity can be measured using a trans-diagnostic self-report instrument. Therefore, the aims of this study were to examine compulsivity in BFRBs using a transdiagnostic measure of compulsivity and to understand the associations of compulsivity with BFRB clinical measures. We hypothesized that compulsivity would correlate with worse symptom severity and higher levels of disability in people with BFRBs.

## Material and Methods

### Participants

Ninety-one adults with DSM-5 TTM or SPD were recruited via advertisements for clinical treatment trials. Inclusion criteria were age 18-65 years and meeting DSM-5 criteria for TTM or SPD. Subjects were excluded if they were unable to give informed consent or were unable to understand/undertake the study procedures. All study procedures were carried out in accordance with the Declaration of Helsinki. The Institutional Review Board of the University of Chicago approved the study and the consent statement. Participants were compensated with a $50 gift card for a local department store.

### Assessments

Participants were interviewed in person and all measures were part of the baseline visit before any treatments were delivered. Participants completed standard diagnostic interviews using the DSM-5 criteria for the diagnosis of TTM and SPD as well as the Mini International Neuropsychiatric Interview 7.0; (Sheehan et al., 1998) for comorbidities. Additionally we assessed basic demographic information, symptom inventories about TTM and SPD, and measures of disability/functioning. Compulsivity was measured using the previously validated 15-item Cambridge-Chicago Compulsivity Trait Scale (CHI-T) which was developed for the rapid and comprehensive measurement of compulsivity (Chamberlain and Grant, 2018).

Other measures included: Massachusetts General Hospital Hair Pulling Scale (MGH-HPS), a 7-item, self-report scale that rates urges to pull hair, actual amount of pulling, perceived control over behavior, and distress associated with hair pulling over the past 7 days (Keuthen et al., 2005), Skin Picking Symptom Assessment Scale (SP-SAS), a 12-item reliable, valid, self-rated scale assessing picking urges, thoughts, and behaviors during the previous 7 days (Grant et al., 2010); Hamilton Depression Rating Scale (HAM-D), a clinician-administered scale which assesses a patient’s level of depression during the past month (Hamilton, 1960); Hamilton Anxiety Rating Scale (HAM-A), a clinician-administered scale which assesses a patient’s level of anxiety during the past month (Hamilton, 1959); Sheehan Disability Scale (SDS), a 3-item self-report measure of psychosocial functioning (reference); Quality of Life Inventory (QoLI), a 16-item, self-report scale that assesses areas of life such as health, love, work, recreation, home, friendships, self-esteem, and standard of living (Frisch et al., 1993); and Barratt Impulsiveness Scale 11th version (BIS), a 30 question self-report measure that is designed to assess various domains of impulsivity, including attentional impulsivity, motor impulsivity, and non-planning impulsivity (Patton et al. 1995).

### Data Analysis

Relationships between CHI-T scores and the other measures of interest were characterized using Spearman’s correlations. For any significant relationships between CHI-T and measures of interest, we then also considered whether Barratt impulsivity scores also related to the measure. This was done since impulsivity and compulsivity may theoretically confound each other in terms of measurement. All analyses were conducted using JMP Pro software and significance was defined as p<0.05.

## Results

The sample comprised n=91 participants, mean (standard deviation) age of 30.9 (10.0) years, 15.9% being male, 78.4% female, and 5.7% non-binary. Of the 91 participants, 48 (54.6%) had TTM, 37 (42.0%) had SPD and 2 (3.4%) had both. 62 individuals (70.4%) had completed college education or higher.

CHI-T total scores correlated with worse disability and quality of life but not with BFRB symptom severity. An overview of correlations between CHI-T scores and the other measures is displayed in Table 1.

Barratt impulsiveness scores (attentional, motor, non-planning) did not relate to quality of life or disability in the sample (all p≥0.1).

## Discussion

This is the first study that we are aware of that has used a validated, trans-diagnostic measure of compulsivity to better understand BFRBs. Our findings were that compulsivity correlates with worse psychosocial functioning (medium effect size) and quality of life (small-medium effect size). This may have relevance for treatment. In the few treatments studies which have assessed psychosocial functioning with the Sheehan Disability Scale, even when BFRB symptoms significantly improved with therapy or medication, disability was found to be unchanged (Falkenstein et al., 2016; Grant et al., 2009; Grant et al., 2016). This may suggest that even promising treatments for BFRBs do not necessarily appear to ameliorate the psychosocial dysfunction associated with BFRBs, which could be contributed to partly by untreated compulsivity. In the current study, we did not find a link between conventional symptom severity measures for BFRBs and disability or quality of life, whereas trans-diagnostic compulsivity did correlate with these clinically important parameters. These findings might suggest the current symptom measures for BFRBs are not including an important aspect of the disease and that a fuller understanding of these symptoms requires measurement of compulsivity. Including validated measures of compulsivity in clinical trials of therapy or medication would also seem to be important for future work.

It is important to note that by ‘compulsivity’ we refer to a broad construct, not specifically to compulsions in OCD. Data indicate that OCD treatments such as selective serotonin reuptake inhibitors (SSRIs) appear to be ineffective for trichotillomania, whereas opioid antagonists or certain glutamate modulators, show promise in clinical trials for trichotillomania (Hoffman et al., 2021). Evaluating effects of such non-SSRI treatments on trans-diagnostic compulsivity in BFRBs would be interesting in future work. Current psychological approaches to trichotillomania often involve habit reversal therapy, which could be regarded as partly targeting compulsivity: again it would be interesting to include a trans-diagnostic compulsivity measure in future clinical trials using such therapies, to examine whether it is ameliorated, or requires a different psychological approach.

Several limitations should be considered. Because this study was conducted in community-recruited participants with TTM and SPD, the findings may not generalize to clinical populations. For example, clinical populations may be expected to have more severe symptoms (on average) and perhaps higher levels of compulsivity than observed herein. Because the study was cross-sectional, direction of effect cannot be shown (i.e. no causal evidence that compulsivity leads to poorer psychosocial functioning and poorer quality of life). Because the aim of the study was to explore links with compulsivity and other variables in people with BFRBs, and due to the nature of the sampling, the study did not have a control group without BFRBs. We do not attempt to conclude that the current findings are specific to BRFBs alone, since confirmation or refutation of that claim would require a different study design. Though a sample size of 91 may be sufficient to detect significant correlations even with moderate effect sizes, follow-up work in larger samples would be valuable, to more rigorously address links between compulsivity and quality of life / functioning.

In conclusion, this study found that compulsivity was associated with higher psychosocial dysfunction and poorer quality of life, in people with BFRBs, to a clinically relevant degree. The data also suggest that compulsivity may be a more important contributor to quality of life in BFRBs than symptom severity; one reason may be that current symptom severity measures do not adequately capture this aspect of the symptoms (i.e. their compulsive nature in some individuals). These results highlight the need to measure compulsivity in those with BFRBs. To what extent these traits may influence response to treatment, however, remains unclear. Given these findings it would also be interesting to explore the contribution of compulsivity to everyday functioning and quality of life in the context of other disorders, as well as whether compulsivity reduces following treatment in other disorders (as well as BFRBs).

## Figures and Tables

**Table 1:** Correlations (Spearman’s rho) between CHI-T scores and variables of interest.

Variable	Rho	p	
Age	0.064	0.5533	
Hamilton Anxiety Rating Scale	0.1779	0.0992	
Hamilton Depression Rating Scale	0.1413	0.1892	
Sheehan Disability Scale	0.2791	0.0085	**
Quality of Life Inventory	-0.2347	0.0278	*
Skin Picking Symptom Assessment Scale	0.1191	0.3487	
Massachusetts General Hospital Hair Pulling Scale	0.138	0.2347	

** significant at p<.01

* significant at p<.05;
